# Acid-mediated reactions under microfluidic conditions: A new strategy for practical synthesis of biofunctional natural products

**DOI:** 10.3762/bjoc.5.40

**Published:** 2009-08-20

**Authors:** Katsunori Tanaka, Koichi Fukase

**Affiliations:** 1 Department of Chemistry, Graduate School of Science, Osaka University, 1-1 Machikaneyama, Toyonaka, Osaka, 560-0043, Japan

**Keywords:** acid-mediated reaction, microreactor, natural products synthesis, oligosaccharide, pristane

## Abstract

Microfluidic conditions were applied to acid-mediated reactions, namely, glycosylation, reductive opening of the benzylidene acetal groups, and dehydration, which are the keys to the practical synthesis of *N*-glycans and the immunostimulating natural product, pristane. A distinctly different reactivity from that in conventional batch stirring was found; the vigorous micromixing of the reactants with the concentrated acids is critical especially for the “fast” reactions to be successful. Such a common feature might be due to the integration of all favorable aspects of microfluidic conditions, i.e., efficient mixing, precise temperature control, and the easy handling of the reactive intermediate by controlling the residence time. The microfluidic reactions cited in this review indicate the need to reinvestigate the traditional or imaginary reactions which have so far been performed and evaluated only in batch apparatus, and therefore they could be recognized as a new strategy in synthesizing natural products of prominent biological activity in a “practical” and a “industrial” manner.

## Introduction

A continuous flow microreactor, an innovative technology, has been used to realize efficient mixing and fast heat transfer in organic syntheses [1–26]. The flow system allows the reaction to be quenched immediately after the formation of the unstable products. Furthermore, once the reaction conditions are optimized for a small-scale operation, the same conditions are directly applicable to large-scale synthesis under the flow process. We have been applying these advantageous features of the microfluidic systems to the “key” but “problematic” acid-mediated reactions under the conventional batch apparatus, in practically preparing bioactive natural products [27–33]. Our successful examples are cation-mediated reactions, such as α-sialylation [28,32], β-mannosylation [31], and reductive opening of the benzylidene acetal groups in sugars [30], for which improved procedure under the microfluidic conditions enabled the preparation of key synthetic intermediates for oligosaccharides on a multi-gram scale, eventually leading to a total synthesis of the asparagine-linked oligosaccharide (*N*-glycan) [32]. A significant improvement has also been achieved for dehydration, which resulted in the industrial scale-synthesis of the immunostimulating natural terpenoid, pristane, of about 500–1000 kg in a year [29]. In this account, we review these acid-mediated reactions and discuss the new aspects of using microfluidic systems for controlling the hitherto difficult reactions in conventional organic synthesis; microfluidic reactions can offer a direct and practical route to the desired compounds without the usual scale-up problems associated with mixing efficiency and the temperature control. They can therefore be regarded as one of the new strategies for the practical synthesis, or in favorable cases, the industrial synthesis of the bioactive natural products.

## Review

### 1. Application of microfluidic systems to the synthesis of asparagine-linked oligosaccharides

**Figure 1:**
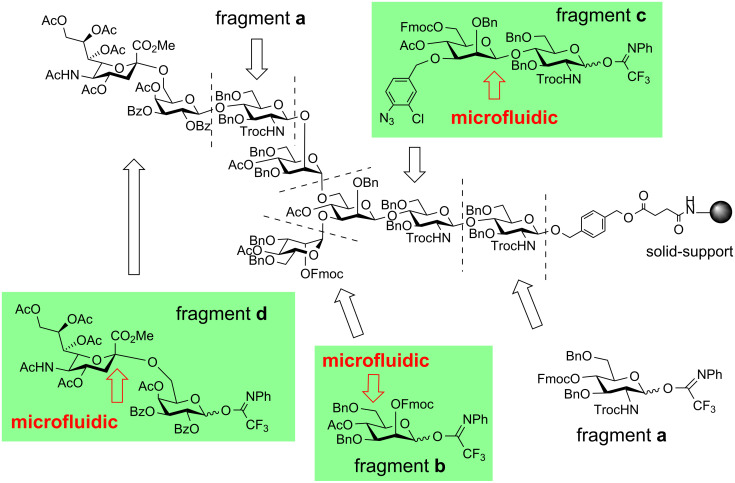
Synthetic strategy for asparagine-linked oligosaccharide on solid support and application of microfluidic systems to fragment synthesis.

Among the various types of oligosaccharide structures, asparagine-linked oligosaccharides (*N*-glycans) are prominent in terms of diversity and complexity [34]. It is becoming clear that they are involved in a variety of important physiological events, such as cell-cell recognition, adhesion, signal transduction, quality control, and circulatory residence of proteins [35–38]. However, isolating large quantities of structurally pure *N*-glycans from natural sources is difficult. Thus, chemical synthesis provides an attractive opportunity to evaluate their biological functions. Although a number of new chemical and/or combined methods employing biological technology have actively been investigated [39–40], an efficient approach to these complex oligosaccharides has yet to be established in terms of (i) selectivity in the glycosyl bond formations, i.e., β-mannosylation and α-sialylation, and (ii) a non-tedious purification process during each step of glycosylation and deprotection. Our interests in elucidating unknown biological functions of mammalian *N*-glycans of the diverse structures, have motivated us to establish a practical and library-directed synthesis of the complex-type *N*-glycans on solid-support [32]; the initial target of our strategy is a sialic acid-containing *N*-glycan with asymmetric branching chains (Figure 1), which is difficult to obtain from natural sources. To prepare the target *N*-glycan as well as other diverse structures of this family in an efficient manner, we designed fragments **a**–**d** with *N*-phenyltrifluoroacetimidate as the leaving group, which can be efficiently glycosylated on solid-support to construct the *N*-glycan structures. Herein, two challenging glycosyl bond formations, i.e., β-mannosylation and α-sialylation, were carried out in advance in solution, by the aid of the microfluidic systems (Figure 1) [28,31–32]. The problem of the key protecting group manipulation, i.e., the reductive opening of benzylidene acetal group, for the large-scale preparation of the fragment **b** [30] could also be circumvented under the microfluidic conditions, as will be discussed in the following sections.

### Microfluidic α-sialylation

We have previously developed an efficient α-sialylation by utilizing the highly reactive sialyl donors having C-5 cyclic imides (Table 1), especially *N*-phthalimide **1a**, by virtue of the “fixed-dipole moment effects” (α-only, 92% on 50 mg scale) [41]. The scale-up in a batch process, however, significantly decreased the yield and selectivity. Thus, a 100 mg scale reaction of **1a** gave only 60% of α-sialoside accompanied by a significant amount of a glycal by-product. The decrease in sialylation efficiency might be due to the high reactivity of the donor **1a**. For such a case, precise reaction control is very difficult under the conventional batch process conditions, especially when the reaction is scaled-up. Thus, the disorder of the reaction factors in the scaled-up batch reaction, i.e., (i) precise temperature control, (ii) mixing efficiency between acceptor, donor, and Lewis acid, and (iii) reaction time, might lead to the glycal production. In order to circumvent these problems, we used a continuous flow microreactor. An application of the microfluidic system to the glycosylation reaction was first reported by Seeberger and co-workers on α-mannosylation [18]. We also have established an efficient microfluidic glycosylation in combination with the affinity separation method [27].

**Table 1:** Optimization of α(2-6)-sialylation using IMM micromixer.

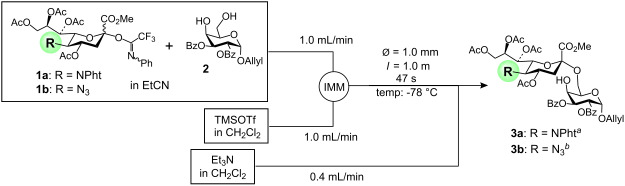					
Entry	Donor **1a**,**b** (M)	Acceptor **2** (M)	TMSOTf (M)	Yield of **3a**,**b** (%)	α : β*^d^*

1	0.15 (**1a**)	0.1	0.08	14*^c^*	α only
2	0.15 (**1a**)	0.1	0.15	88	α only
3	0.2 (**1a**)	0.1	0.15	>99	α only
4	0.2 (**1b**)	0.1	0.15	>99	20 : 1

*^a^*Batch results: 92%, α-only on 50 mg scale; 60%, α : β = 97 : 3 on 100 mg scale. *^b^*Batch results: 90%, α : β = 9 : 1 on 100 mg scale. *^c^*Mainly, glycal derived from **1a** and acceptor **2** were recovered by TLC analysis. *^d^*Based on ^1^H NMR analysis.

For the present microfluidic sialylation, a propionitrile solution of sialyl donor **1a** and acceptor **2** with various concentrations was mixed with TMSOTf solution in dichloromethane at −78 °C using an IMM micromixer [42] with a channel width of 40 μm at a flow rate of 1.0 mL/min (Table 1). After the reaction mixture was allowed to flow at −78 °C for an additional 47 seconds through a reactor tube (Ø = 1.0 mm, *l* = 1.0 m), the mixture was quenched by another flow of excess triethylamine dissolved in dichloromethane by using a T-shaped mixer at −78 °C. When the concentrations of the donor **1a**, acceptor **2**, and TMSOTf were adjusted to 0.15 M, 0.1 M, and 0.08 M, respectively, disaccharide **3a** was obtained in only 14% yield and a large amount of the acceptor **2** was recovered (entry 1). However, we were pleased to find that the yield of **3a** dramatically increased (88%) when the concentration of the Lewis acid was increased to 0.15 M (entry 2). Finally, the desired α-sialoside **3a** was obtained in quantitative yield by increasing the concentration of the donor **1a** to 0.2 M (entry 3). Thus, the microfluidic reaction successfully controlled the high reactivity of the sialyl donor **1a** for α-sialylation. Obviously, vigorous and rapid mixing of the substrates with the high concentrations of the acid is responsible for the success of the microfluidic sialylation; the trend is completely different from that of the corresponding batch reaction, since the decomposition of the donor has long been regarded as a severe problem under such drastic conditions [41]. The use of excess amounts of donor **1a** and/or TMSOTf in batch reaction did not improve the sialylation yield, but rather resulted in a large amount of glycal production. Apparently, the efficient micromixing between substrates and the high concentration of the acid should accelerate the reaction, while the rapid heat transfer prevented the undesired hydrolysis and glycal formation. The “flow system” also enabled the residence time to be controlled before the decomposition of sialoside **3a** under the strongly acidic conditions. This new aspect under microfluidic conditions was found to be general to the acid-mediated reactions, and similar results can be seen in the following sections.

Unfortunately, the selective deprotection of the *N*-phthalyl group in the presence of the C-1 methoxycarbonyl in **3a** was troublesome on the 1–2 g reaction scale. As an alternative to the *N*-phthalyl function, we also employed the C-5 azide group in sialyl donor **1b** because this azide group should direct similar “fixed-dipole moment effects”, but should be easier to convert to naturally occurring *N*-substituents of neuraminic acids, i.e., *N*-acetyl or *N*-glycolyl groups (see structure in Figure 1) [32]. As anticipated, sialylation between **1b** and galactosyl acceptor **2** in the presence of TMSOTf as an activator and 4Å molecular sieves in propionitrile provided **3b** in 90% yield with good α-selectivity (α : β = 9 : 1 on 100 mg scale). Furthermore, applying the continuous microfluidic sialylation, which was established for the *N*-phthalyl derivative **1a** (Table 1, entry 3), improved both the yield and α-selectivity (entry 4): **3b** was obtained quantitatively with near perfect α-selectivity (α : β = 20 : 1). The α and β stereoisomers were easily separated by chromatography on silica-gel, and the pure α-isomer was readily converted to the desired imidate fragment **d** (Figure 1) using the general procedure. Hence, we successfully prepared fragments **d** on the 5–10 g scale [32].

### Microfluidic β-mannosylation

Stereoselective formation of the β-mannoside linkage, a key glycosylation in the synthesis of the βMan(1→4)GlcNTroc fragment **c** of *N*-linked glycans, is another challenging topic in oligosaccharide synthesis. Various methods, such as intramolecular aglycon delivery (IAD) or glycosylation with 4,6-*O*-benzylideneacetal-protected α-mannosyltriflates, have recently been reported and successfully applied to β-mannoside synthesis [31,39–40,43–45]. We also have achieved excellent β-selectivity in the reaction of 4,6-*O*-benzylidenemannopyranosyl-*N*-phenyltrifluoroacetimidate (**4**) with *N*-Troc-glucosamine acceptor **5** (R = Bn, 93% yield, β : α = 95 : 5 on 20 mg scale) using the bulky and dual Lewis acid/cation trap reagent, TMSB(C_6_F_5_)_4_ (Table 2, entry 1) [46–47].

**Table 2:** β-Mannosylation under batch conditions.

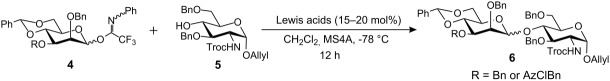					
Entry*^a^*	Lewis acids	Addition of LA	scale (mg)	Yield (%)*^b^*	β : α*^c^*

1	TMSB(C_6_F_5_)_4_	dropwise	20	88	95 : 5
2	TMSOTf	dropwise	20	84	93 : 7
3	TMSOTf	dropwise	50	63*^d^*	NA
4	TMSOTf	dropwise	500	27*^d^*	NA
5	TMSOTf	in one portion	900	61*^d^*	4.9 : 1

*^a^*Reaction is performed using 1.5 equiv of donor **4** relative to acceptor **5**. *^b^*Isolated yields for β-isomer. *^c^*Based on ^1^H NMR analysis. *^d^*Additionally, acceptor **5**, its silylated derivative, and decomposed products of **4** were obtained by TLC analysis.

Nevertheless, it is difficult to apply our β-mannosylation protocol to a few gram-scale synthesis of the βMan(1→4)GlcNTroc fragment **c** because the scaled-up glycosylation requires a large quantity of the bulky TMSB(C_6_F_5_)_4_ activator, which has limited commercial availability [46–47]. Therefore, from a practical viewpoint for preparing the fragment **c** as a starting material, we refocused on applying the more common TMSOTf as a glycosyl activator because our earlier experiments indicated that TMSOTf shows a good yield and β-selectivity on a 20 mg scale (90% yield, β : α = 93 : 7) (entry 2) [46]. However, the efficiency of glycosylation catalyzed by TMSOTf is extremely sensitive to the reaction scale as well as the addition speed of the Lewis acid (entries 2–5). When TMSOTf was added dropwise to a solution of mannosyl donor **4** and acceptor **5** at −78 °C, the yield of β-mannoside **6** gradually decreased as the reaction scale increased (entries 2–4). On a 50 mg scale, 63% of β-disaccharide **6** was isolated, whereas only 27% of β-isomer **6** was obtained on a 500 mg scale (entries 3 and 4). For an unknown reason, slow addition of a Lewis acid in the larger scale reactions inhibited the glycosylation process at an earlier stage [31]. Moreover, even the subsequent addition of the TMSOTf catalyst did not activate the glycosylation between the remaining starting materials. These results cannot be clearly explained based on presently available data. On the other hand, when the acid was added to the initial solution of **4** and **5** in one portion, mannosylation proceeded smoothly (entry 5). However, the β-selectivity decreased to 4.9 : 1, presumably due to the exothermic nature of the reaction, i.e., heat is generated while rapidly mixing, which leads to an overall decrease in the isolated yield of β-disaccharide **6** (61% on 900 mg scale). Therefore, we decided to examine the microfluidic conditions based on the observations shown in Table 2, which indicate that the success of the current glycosylation depends on the fast addition and the efficient mixing with the Lewis acid to produce the active intermediates at the low temperature, which subsequently and slowly reacted with the acceptor **5** in batch apparatus.

**Figure 2:**
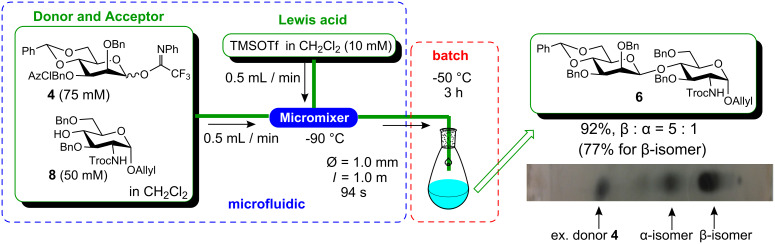
β-Mannosylation using an integrated microfluidic/batch system. Yield and β/α-ratio are analyzed by ^1^H NMR and HPLC.

We initially constructed the microfluidic system shown in Figure 2, based on our previous experiences with microfluidic α-sialylation [28,32]. In addition to the aspects mentioned above, an attractive feature of the microfluidic reaction is that the reaction can be readily optimized under the flow process [18]; the optimal conditions, i.e., concentrations of the substrates, mixing speed, temperature, and residence time, are rapidly determined using a small quantity of materials. For this optimization, we have used the Comet X-01 micromixer [48] with a channel width of ca. 500 μm, where the micromixing with IMM mixer (40 μm) caused the significant solution blockage problems due to the low solubility of both the donor **4** and the acceptor **5** in dichloromethane at very low temperature of −78 to −90 °C. Rapid screening of more than 30 conditions in a combinatorial fashion led to a new reaction system where the microfluidic system is integrated with a conventional batch apparatus (Figure 2) [31]. Namely, the reaction solution resulting from efficient micromixing between the reactants at a low temperature, was subsequently inserted into the batch system, and then was conventionally stirred in a flask for a few hours to complete the reaction. Although it is theoretically possible to maintain an indefinite residence time by increasing the reactor tube length, under certain conditions, i.e., when the reaction has to proceed for more than an hour, employing an extremely long tube is impractical. The optimal conditions in the integrated microfluidic/batch apparatus as depicted in Figure 2, i.e., micromixing at −90 °C and a batch reaction at −50 °C for 3 h, provided α/β-mannoside **6** (R = Bn) in 92% yield and with a moderate β-selectivity (β : α = 5.0 : 1). Under the established conditions, mannosylation proceeds gradually in the flask, by TLC analysis. It should be noted that although the β-selectivity was somewhat lower than that observed in the small-scale batch reaction (Table 2, entry 2), the isolated β-mannoside **6** could be obtained with similar efficiency (77% for microfluidic reaction versus 84% for 20 mg scale batch reaction) [31]. Moreover, under the established conditions, the compound **6** was reproducibly obtained even in the scaled-up synthesis by simply preparing stock solutions of substrates and reagents, and then continuously pumping them into the integrated microfluidic/batch system. Thus, the reproducibility and scaled-up preparation of **6**, and hence the fragment **c**, is noteworthy from the viewpoint of preparing an important synthetic intermediate for complex *N*-glycans.

### Microfluidic reductive opening of sugar 4,6-*O*-benzylidene acetals

The microfluidic conditions can also be applied to an efficient procedure for the reductive opening of 4,6-*O*-benzylidene acetals [30], a key functional group manipulation toward fragment **b** for our *N*-glycan synthesis. Reductive opening of sugar 4,6-*O*-benzylidene acetals by the combination of acid/hydride reagents is one of the most useful transformations in the field of carbohydrate chemistry [49], not only for our current *N*-glycan case, since the benzyl-protected derivatives at either the C4- or C6-hydroxyl can be selectively prepared by the choice of reagent and/or solvent systems [50–51].

The typical procedure of the reductive opening of the acetals involves the very slow addition of the acid to a mixture of the substrate and excess hydride reagent at 0 °C, followed by continuous stirring at room temperature for another few hours to ensure the completion of the reaction. Since the reaction is exothermic, it is important to control the addition speed of the acid in order to prevent concomitant acid-catalyzed hydrolysis of the benzylidene groups. However, the yields are not always reproducible, especially when the reaction is performed in a large scale, giving rise to the hydrolyzed by-products, 4,6-*O*-diols. The precise temperature control and the mixing efficiency between the substrate, acid, and hydride source are critical for this transformation. In order to facilitate the synthetic procedure, therefore, we used a continuous flow microreactor.

Optimal conditions for reductive opening of 4,6-*O*-benzylidene acetals by a microfluidic system, similar to the α-sialylation and the β-mannosylation protocols, were examined using the glucose 4,6-*O*-benzylidene acetal **7** (Table 3). Benzylidene acetal **7** (0.1 M) and Et_3_SiH (1.0 M) dissolved in CH_2_Cl_2_ were mixed with various concentrations of BF_3_·OEt_2_ in CH_2_Cl_2_ (0.1–1.0 M) at 0 °C using a Comet X-01 micromixer [48] at the flow rate of 0.5 mL/min. After the reaction mixture was allowed to flow at room temperature for approximately 90 seconds through a reactor tube (Ø = 1.0 mm, *l* = 0.9 m), the mixture was quenched by saturated NaHCO_3_ solution at 0 °C.

**Table 3:** Reductive opening of benzylidene acetals under microfluidic conditions.

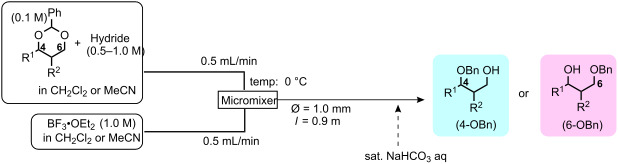						
Entry	Substrate	Reducing agent	Solvent	Product	Yield (%, microfluidic)*^a^*	Yield (%, batch)*^a,b^*

1	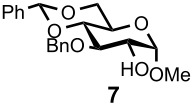	Et_3_SiH (1.0M)	CH_2_Cl_2_	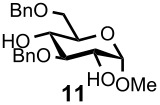	93*^c^* (6-OBn)	58
2	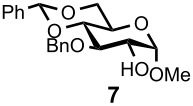	BH_3_·Et_2_NH (0.5M)	CH_2_Cl_2_	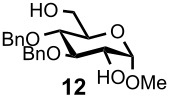	100 (4-OBn)	90
3	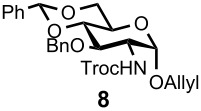	Et_3_SiH (1.0M)	CH_2_Cl_2_	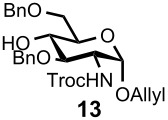	91 (6-OBn)	83
4	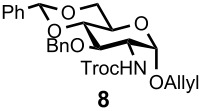	BH_3_·Et_2_NH (0.5M)	CH_2_Cl_2_	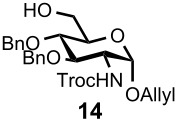	100 (4-OBn)	86
5	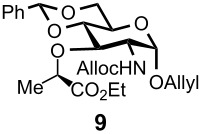	BH_3_·Et_3_N (0.5M)	MeCN	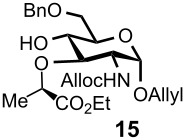	100 (6-OBn)	NA*^d^*
6	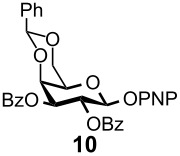	Et_3_SiH (1.0M)	CH_2_Cl_2_	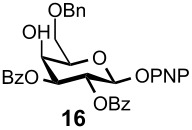	91 (6-OBn)	62

*^a^*Isolated yields. *^b^*Reaction was performed at 100–500 mg scale. *^c^*4-*O*-Benzyl derivative was obtained in 5% yield. *^d^*60–70% Yields for the case of corresponding *N*-Troc derivative. *^e^*PNP: *p*-nitrophenol.

When 0.1 M solution of BF_3_·OEt_2_ in CH_2_Cl_2_ was used, only a hydrolyzed compound was obtained in about 50% yield accompanied with recovery of the starting material **7**. However, the yield of 6-*O*-benzyl derivative **11** dramatically increased when the concentration of BF_3_·OEt_2_ was increased up to 1.0 M; the desired **11** was obtained in 93% yield together with 5% of the 4-*O*-benzyl derivative **12**, and no hydrolyzed by-product was detected (Table 3, entry 1). Surprisingly, the reaction with a more concentrated BF_3_·OEt_2_ solution (BF_3_·OEt_2_ : CH_2_Cl_2_ = 1 : 1) also provided **11** in quantitative yield. The reaction is very rapid and **11** was obtained within a minute after mixing with the acid in the micromixer device. These results are in marked contrast to those of the batch reaction, which requires dilution of the Lewis acid and longer reaction time in order to keep the hydrolysis to a minimum. Very similar to the microfluidic α-sialylation described above, the rapid and vigorous mixing with the highly concentrated acid was critical for the acid-mediated microfluidic reaction to be successful.

Gratifyingly, by applying the established conditions above, the glucose, glucosamine, and galactose 4,6-*O*-benzylidene acetals **7**–**10** were selectively transformed into the corresponding 4- or 6-*O*-benzyl derivatives **12**–**16** in nearly quantitative yields (entries 2–6). Thus, the treatment of **7** with BH_3_·Et_2_NH selectively provided 4-*O*-benzyl derivative **12** in quantitative yield (entry 2). The benzylidene group of glucosamine derivative **8** was also selectively cleaved under the microfluidic conditions by utilizing either Et_3_SiH or BH_3_·Et_2_NH as a hydride source to provide the 6- and 4-*O*-benzyl derivatives **13** and **14** in excellent yields, respectively (entries 3 and 4). The BH_3_·Et_3_N in MeCN system was also applicable to the microflow reduction of muramic acid derivative **9**, which contains a lactic acid ester at the C3-hydroxyl, affording 6-*O*-benzyl derivative **15** as a single isomer (entry 5). Finally, the selective reduction of galactose derivative **10** gave the 6-*O*-benzyl derivative **16** in 91% yield (entry 6). It is worthwhile mentioning that the established microfluidic reaction reproducibly provided the *O*-benzylated compounds **11**–**16** in greater than 90% yields in all cases, out of the three experiments performed in each entry of Table 3. Furthermore, no special dehydration procedures, such as pre-drying of the reaction apparatus and the solvents by molecular seives, are necessary, making the present microflow reaction a practical procedure for large-scale synthesis [30]. Based on the established microfluidic protocol, fragment **b** for the *N*-glycan synthesis, was continuously and reproducibly obtained even on a 10-gram scale [32].

The successful application of the microfluidic systems to the three hitherto “difficult” acid-mediated reactions under the conventional batch conditions, namely, α-sialylation, β-mannosylation, as well as reductive opening of sugar 4,6-*O*-benzylidene acetals, significantly facilitated the large scale preparation of the *N*-glycan fragments **b**–**d**. A stock of these key synthetic intermediates eventually led to the first solid-supported synthesis of the sialic acid-containing complex-type *N*-glycan (Figure 1) [32].

### 2. Microfluidic dehydration: a process synthesis of immunoactivating natural product, pristane

2,6,10,14-Tetramethylpentadecane (pristane) is a saturated isoprenoid isolated from the basking shark, *Cetorhinus maximus* [52–56]. This hydrocarbon oil is known to induce tumorigenesis in mice and arthritis and lupus nephritis in rats, and has been widely used as an adjuvant for monoclonal antibody production in mouse ascites [57–59]. However, in 2002, the basking shark was listed on Article II of the Washington Convention (Convention on International Trade in Endangered Species of Wild Fauna and Flora), and since then, the availability of pristane from a natural source became very limited. Therefore, an efficient chemical synthesis of pristane has been long desired.

**Scheme 1:**
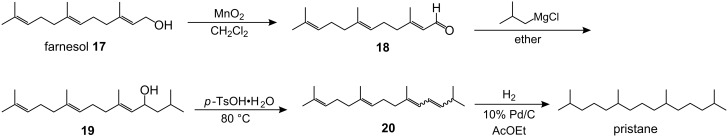
Synthesis of pristane.

When considering the synthesis of this simple hydrocarbon in a non-stereoselective manner, one can immediately come up with a commonplace route, i.e., oxidation of farnesol **17**, alkylation, dehydration, and hydrogenation, as shown in Scheme 1. The synthesis is quite simple when 50 mg of the sample is prepared, but it suddenly becomes difficult when 200 kg of this hydrocarbon is required in a year with more than 98% purity. Namely, the preparation of 5 kg of pristane in about a week is necessary in order to supply enough material in the market.

The challenging step in Scheme 1 is the acid-catalyzed dehydration of allylic alcohol **19**. When the reaction was performed in 100 mg scale using a catalytic amount of *p*-TsOH in benzene at 80 °C, the corresponding diene **20** was obtained in 55% yield as its (*E*)- and (*Z*)-stereoisomers, which was transformed to pristane by hydrogenation. However, when the scale was raised to 100 g, various cation-mediated by-products, such as the cyclized products or the alkyl group-migrated compounds were produced. As expected, these hydrocarbons were very difficult to separate from the desired diene **20**, even by repeated distillation or by silica gel chromatography, although the latter is not realistic for kilogram-scale purification.

**Figure 3:**
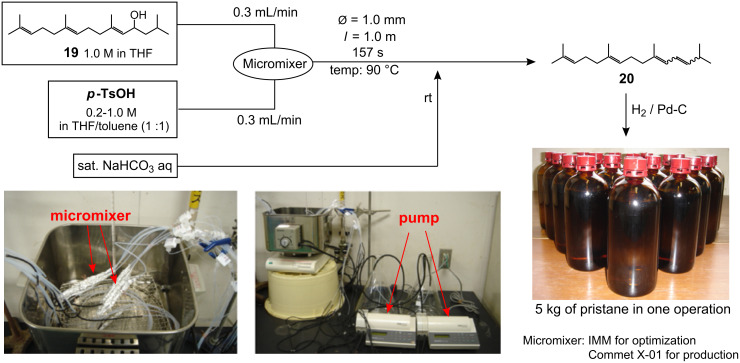
Process synthesis of pristane via microfluidic dehydration as a key step.

Inspired by the successful application of the microfluidic systems to the acid-mediated reactions during the *N*-glycan synthesis described above, we examined the microfluidic dehydration under the following conditions (Figure 3) [29]: Allylic alcohol **19** (1.0 M in THF) was mixed with a solution of *p*-TsOH (various concentrations of 0.2–1.0 M in THF : toluene = 1 : 1) at 90 °C using an IMM micromixer [42] at each flow rate of 0.3 mL/min. After the reaction mixture was allowed to flow for additional 157 seconds through a reactor tube (Ø = 1.0 mm, *l* = 1.0 m) at 90 °C, the mixture was quenched by a saturated NaHCO_3_ solution at room temperature.

When 0.2 M solution of *p*-TsOH was used, only a trace amount of **20** was obtained and the starting material **19** was largely recovered. However, we again found that the yield of the dehydrated compound depends on the concentration of the acid; **20** was finally obtained in 80% yield (total yield from farnesol **17**) at an acid concentration of 1.0 M. The common features observed for the microfluidic α-sialylation and the reductive opening of the benzylidene acetals, and opposed to the conventional batch reactions, can also be applied to the current dehydration; namely, the success of “very fast” acid-mediated reactions under the microfluidic conditions owes to the rapid micromixing with the concentrated acid. It is noted that under the established microfluidic conditions, the formation of other by-products could not be detected by TLC analysis.

Having established the optimal conditions for dehydration, the kilogram synthesis of pristane was examined (Scheme 1 and Figure 3) [29]. The crude alcohol **19**, derived from 8 kg of farnesol **17** without any purification process, was subjected to the key microfluidic dehydration under the conditions established in Figure 3. For such a large-scale microfluidic reaction, we again introduced Comet X-01 [48], which we know from the previous examples, exhibited similar mixing efficiency to the IMM micromixer and avoids the blocking problem by using the relatively large tube hole (ca. 500 μm). This is especially useful for a synthetic process in which an easily crystallized material, such as TsOH, is used at high concentration. Therefore, this mixer, together with its availability at a low price, is well suited for establishing a micro chemical plant. As shown in Figure 3, we arranged 10 micromixers in a row and achieved the eight kilogram-scale dehydration during 3–4 days. It is noted that the present micro chemical plant does not require any special apparatus or devices and such a simple system in Figure 3 continuously performed efficient dehydration. The resulting solution eluted from the micromixing system was quenched with a saturated NaHCO_3_ solution, extracted with ethyl acetate, concentrated, and the mixtures were shortly passed through a silica gel pad in order to remove the hydrophilic by-products, affording the pure diene **20**.

Finally, the hydrogenation provided pristane in 50–55% overall yields (ca. 5 kg) with >99% purity based on gas chromatography analysis. Since the present pristane synthesis involves only one simple purification step by filtration with a silica gel pad, we believe that our protocol is superior to the hitherto known synthesis, which involves purification by tedious multiple distillation at the final stage. Indeed, the synthetic pristane formed by this route is confirmed to induce antibody production in mouse ascites twice as efficiently as natural pristane or the other synthetic products, due to non-negligible contamination of the other hydrophobic compounds [52–56].

**Scheme 2:**
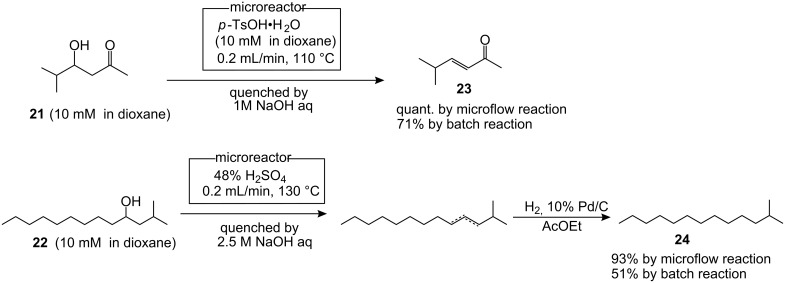
Microfluidic dehydration.

In order to evaluate the efficiency of dehydration under the microfluidic conditions, the reactivity of β-hydroxyketone **21** and alkanol **22** were also tested (Scheme 2) [29]. Gratifyingly, both substrates provided the corresponding dehydrated products **23** and **24** in almost quantitative yields under the conditions established in Figure 3. For both cases, the conventional batch reaction gave lower yields of the products due to recovery of the starting materials and formation of other by-products. Therefore, an efficient and general protocol for dehydration was realized by using the micromixing system.

## Conclusions

In conclusion, we have achieved efficient glycosylation, reductive opening of the benzylidene acetal groups, and dehydration under microfluidic conditions. During our research in applying the microfluidic systems to these acid-mediated reactions, a distinctly different reactivity from that in the conventional batch stirring was found: vigorous micromixing of the reactants with the concentrated acids is critical especially for the “fast” reactions to be successful. Such a common feature might be due to the integration of all favorable aspects of microfluidic conditions, namely, efficient mixing, precise temperature control, and the easy handling of the reactive intermediate by controlling the residence time. As can be seen from the three examples cited in this review, rapid determination of the reaction conditions is another aspect of using the microfluidic conditions in a combinatorial fashion. The efficiency of the acid-mediated microfluidic reactions cited in this review, together with the other successful flow reactions reported by us and the other researchers, indicate the need to reinvestigate the traditional or imaginary reactions which have so far been performed and evaluated only in batch apparatus, and therefore have not been widely utilized for organic synthesis. In other words, such reactions could be refocused as “new generation reactions”. The authors strongly believe that the microfluidic reactions could be recognized as a new strategy for the synthesis of natural products of the prominent biological activity in a “practical” and a “industrial” manner.
